# HIV Gag and PR: partners in resistance to protease and maturation inhibitors

**DOI:** 10.1186/1742-4690-8-S2-O40

**Published:** 2011-10-03

**Authors:** Monique Nijhuis

**Affiliations:** 1 Dept of Medical Microbiology, Virology, University Medical Center Utrecht, The Netherlands

## 

Maturation is an essential step in the HIV-1 life-cycle. It is the transition of immature, non-infectious virus particles to mature and infectious virions triggered by the proteolytic cleavage of the precursor Gag and GagPol polyproteins by the viral enzyme protease.

HIV-1 protease recognizes the asymmetric shape of the peptide substrates, rather than a particular amino acid sequence. These peptides have a super-ímposable secondary structure, yielding a substrate "envelope" which fits within the protease substrate-binding region. There are however, a few differences between the substrates in which amino acid side-chains protrude out of the "envelope" which contribute to the different rates of cleavage. Detailed knowledge of the structure of HIV protease and its substrate has led to the development of specific protease inhibitors (Pls), which have played a major role in antiviral therapy. They bind protease with high affinity but tend to occupy more space than the natural substrates. Resistance to PI is usually a stepwise process in which a substitution in the substrate-binding cleft of the protease is observed first. This results in an overall enlargement of the catalytic site and decreased binding to the inhibitor and, in parallel, to some decrease in binding to the natural substrate and to decreased viral replication. Several studies have identified an association between selection of protease mutations and mutations in the Gag substrate. We have shown that substitutions in the NC/p1 cleavage site alone, without any alterations in the viral protease were selected during *in vitro* PI exposure. These NC/ p1 changes conferred PI resistance, which could directly be related to increased gag processing. A detailed analysis of clinical isolates of patients on PI therapy also indicated that NC/p1 mutations strongly contribute to PI resistance besides compensating for a loss in viral replication.

A novel class of maturation inhibitors target the gag structural proteins instead of the viral protease. Bevirimat (BVM; PA-457, MPC-4326) was the first of these maturation inhibitors to go into clinical trials. The CA/p2 cleavage site has been identified as the bevirimat target region by western-blotting and *in vitro* resistance selection studies. Clinical studies (phase 2b) showed that baseline polymorphisms slightly downstream of the CA/p2 cleavage site, known as the QVT-motif also confer resistance. We have shown that bevirimat resistance mutations occur more frequently in PI resistant isolates compared to isolates from PI treatment naive individuals and this could mainly be attributed to mutations in the QVT-motif. Furthermore, we have shown that PI resistance mutations alter the bevirimat resistance profiles selected *in vitro* and impact the level of bevirimat resistance.

All these findings signify the strong interaction between the viral protease and its substrate suggesting that PI therapy can have important consequences for future treatment with maturation inhibitors.

